# Rapid Antibody-Based COVID-19 Mass Surveillance: Relevance, Challenges, and Prospects in a Pandemic and Post-Pandemic World

**DOI:** 10.3390/jcm9103372

**Published:** 2020-10-21

**Authors:** Robin Augustine, Suvarthi Das, Anwarul Hasan, Abhilash S, Shaheen Abdul Salam, Priya Augustine, Yogesh Bharat Dalvi, Ruby Varghese, Rosita Primavera, Hadi Mohamad Yassine, Avnesh S. Thakor, Bhavesh D. Kevadiya

**Affiliations:** 1 Department of Mechanical and Industrial Engineering, College of Engineering, Qatar University, Doha PO Box 2713, Qatar; robin.augustine@qu.edu.qa; 2 Biomedical Research Center (BRC), Qatar University, Doha PO Box 2713, Qatar; hyassine@qu.edu.qa; 3 Department of Medicine, Stanford University Medical Center, Palo Alto, CA 94304, USA; suvarthi.d@gmail.com; 4 Department of Microbiology, Majlis Arts and Science College, Puramannur, Malappuram, Kerala 676552, India; abicool1986@gmail.com; 5 Department of Biosciences, MES College Marampally, Aluva, Ernakulam, Kerala 683107, India; shaheensalam121@gmail.com; 6 Department of Zoology, Providence Women’s College, Kozhikode, Kerala 673009, India; priyaaugustine2000@gmail.com; 7 Pushpagiri Research Centre, Pushpagiri Institute of Medical Science & Research, Tiruvalla, Kerala 689101, India; yogesh.botany@gmail.com (Y.B.D.); rubyvrghs26@gmail.com (R.V.); 8 Interventional Regenerative Medicine and Imaging Laboratory, Department of Radiology, School of Medicine, Stanford University, Palo Alto, CA 94304, USA; primaverarosita@gmail.com (R.P.); asthakor@stanford.edu (A.S.T.); bbhaveshpatel@gmail.com (B.D.K.)

**Keywords:** antibody, immunoglobulin, COVID-19, SARS-CoV-2, Point of Care Test, PoCT, population surveillance

## Abstract

The aggressive outbreak of the severe acute respiratory syndrome coronavirus-2 (SARS-CoV-2) as COVID-19 (coronavirus disease-2019) pandemic demands rapid and simplified testing tools for its effective management. Increased mass testing and surveillance are crucial for controlling the disease spread, obtaining better pandemic statistics, and developing realistic epidemiological models. Despite the advantages of nucleic acid- and antigen-based tests such as accuracy, specificity, and non-invasive approaches of sample collection, they can only detect active infections. Antibodies (immunoglobulins) are produced by the host immune system within a few days after infection and persist in the blood for at least several weeks after infection resolution. Antibody-based tests have provided a substitute and effective method of ultra-rapid detection for multiple contagious disease outbreaks in the past, including viral diseases such as SARS (severe acute respiratory syndrome) and MERS (Middle East respiratory syndrome). Thus, although not highly suitable for early diagnosis, antibody-based methods can be utilized to detect past infections hidden in the population, including asymptomatic ones. In an active community spread scenario of a disease that can provide a bigger window for mass detections and a practical approach for continuous surveillance. These factors encouraged researchers to investigate means of improving antibody-based rapid tests and employ them as reliable, reproducible, sensitive, specific, and economic tools for COVID-19 mass testing and surveillance. The development and integration of such immunoglobulin-based tests can transform the pandemic diagnosis by moving the same out of the clinics and laboratories into community testing sites and homes. This review discusses the principle, technology, and strategies being used in antibody-based testing at present. It also underlines the immense prospect of immunoglobulin-based testing and the efficacy of repeated planned deployment in pandemic management and post-pandemic sustainable screenings globally.

## 1. Introduction

The outbreak of coronavirus disease-2019 (COVID-19) has rapidly spanned the world, with over 39 million reported cases and 1 million deaths (as of 16 October 2020) across multiple countries [1]. The severity of the disease in diagnosed patients has broad variations ranging from asymptomatic carriers, or mild cases needing supportive care, to severe cases requiring extracorporeal membrane oxygenation [2]. The patients with symptom onset are reported to be most contagious and to shed the virus [3]. However, as clinical testing capacities increase, numbers of asymptomatic or pre-symptomatic cases keep skyrocketing. This jeopardizes proper screening and transmission-control. This especially poses a serious challenge since governments and regulatory bodies relax stay-at-home orders and reopen partially. In some cases, decision-makers are being forced to revert their reopening plans on account of increasing case numbers. Surveillance and largescale accessible diagnostics always play an important part to curb the spread of infectious pathogens and reduce mortality during an outbreak such as this [4,5]. During past outbreaks, the lack of a rapid and simple serological test limited past infection tracing and assessment of overall health impacts. A reliable immunoglobulin (antibody)-based surveillance plan that can detect even past and non-active infections can help understand community spread dynamics and the level of susceptibility in a specific population or region [6]. These tests detect the presence of specific proteins called antibodies or immunoglobulins produced in response to antigen/s of the pathogen [7]. Moreover, antibody-based diagnostic tests at mass scale can help gather epidemiological data on infected cases, and track them for possible COVID-19 associated complications such as cardiovascular [8,9,10,11], neurological [12,13,14,15], and other unknown pathophysiological conditions [16]. 

**Figure 1:**
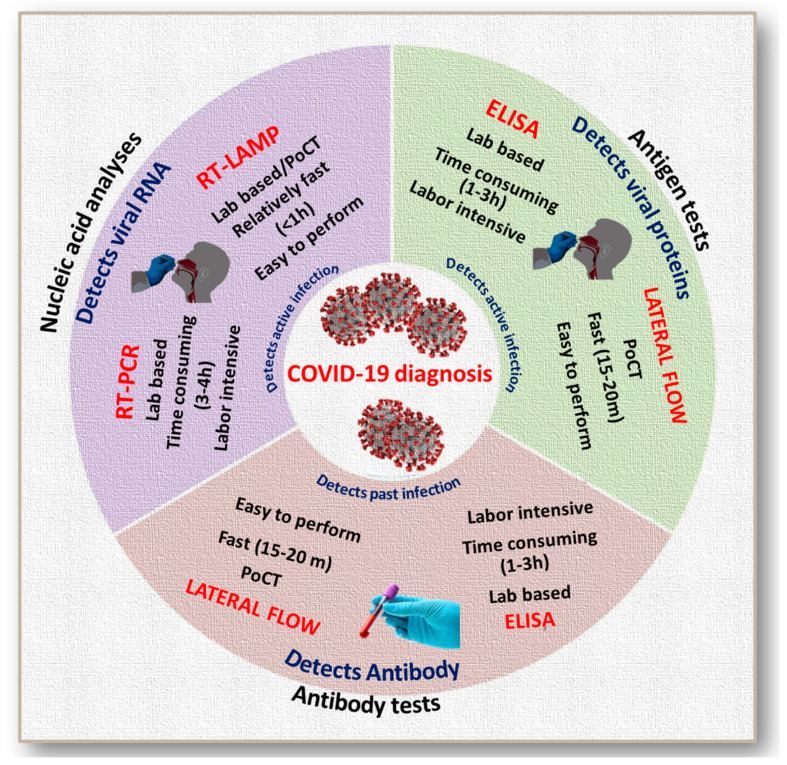
Scheme showing the comparison of different methods used for COVID-19 diagnosis (PoCT: Point of Care Test). Nucleic acid tests detect the genetic material from the virus. To collect the infected cells, a nasal or throat swab of the patient is required. Through a series of chemical reactions, copies of viral genetic material are produced. It helps to analyze whether or not a person has active infection. Polymerase chain reaction (RT-PCR) and Reverse Transcription Loop-Mediated Isothermal Amplification (RT-LAMP) are two major tests used in nucleic acid analysis. In total, 3–4 h are required to carry out RT-PCR. RT-LAMP is a simple method which can detect few copies of target nuclei sequences under isothermal conditions with the help of specially designed additional primer sets. It can be performed in laboratories or tested in point of care settings. RT-LAMP reaction can be completed within an hour. A rapid antigen test is a rapid diagnostic test which detects the presence of viral antigen usually in the nasopharyngeal samples. This is the newest method among the above described tests. In this type of test, chemicals fragment the virus and then antibodies attached to a plate detect these fragments. This provides relatively fast test results within 15 min. This again, helps to analyze whether or not a person has active infection. Lateral flow and enzyme-linked immunosorbent assays (ELISA) are the major types of antigen tests. Antibody tests help to detect past infections or those at advanced stages. Lateral flow test is the commonly accepted cost-effective antibody test which does not require any specialized equipment. It is a relatively fast method of testing, completed within 15–20 min. This type of assay is performed on blood samples. ELISA is a laboratory-based antibody test which requires skilled professionals and is a time-consuming task of 1–3 h. By this method, up to 96 samples can be tested per batch.

To date, there are several strategies for diagnosing COVID-19, which are mainly based on viral nucleic acid [17,18,19,20] or antigen detection [21], and detection of the host’s immunological responses [22]. Figure 1 illustrates the most widely used diagnostic tests for COVID-19. All these tests have their own advantages and shortcomings. Real-time polymerase chain reaction (RT-PCR), despite its unbeatable accuracy and precision, has limitations such as the requirement of highly trained technicians, laboratory equipment, and/or high expense and time required per test [23]. The shortage or unavailability of reagents for RNA extraction from the virus is another challenge [23]. Unlike RT-PCR, emerging reverse transcription loop-mediated isothermal amplification (RT-LAMP) can complete the reactions within an hour, with limited resources, and without highly skilled technicians. However, the efficacy of RT-LAMP, just like other molecular diagnostic methods such as recombinase polymerase amplification (RPA) and clustered regularly interspaced short palindromic repeats-based (CRISPR-based) detection, is largely limited by the precision in primer designs and specificity of other tools [24]. Enzyme-linked immunoassay (ELISA)-based antigen or antibody detection is also limited by lengthy protocols and the requirement of expensive lab equipment. Antigen tests are recently being introduced as a rapid diagnostic tool to screen community spread of the pandemic [25]. Limit of Detection (LoD) for antigen tests tend to be higher than that of nucleic acid amplification (NAA) [26]. Despite the questions regarding the sensitivity, among the currently available methods, lateral flow antigen tests are the fastest and can be performed by any healthcare professional without additional training [27]. 

Both NAA and antigen-based tests can only diagnose currently active cases with significant viral shedding. Antibody-based tests, on the other hand, can trace past infections. They are cheap, uncomplicated, faster than RT-PCR (the universally accepted gold-standard for coronavirus diagnosis), and can be deployed in remote areas. The testing tools can be easily acquired and standardized [28,29]. There are also published studies validating serological tests, including virus-specific IgM (Immunoglobulin M) and IgG (Immunoglobulin G), during past viral epidemics such as SARS (severe acute respiratory syndrome) [30,31]. An antibody-based test with a reliable threshold value of specificity and sensitivity, when performed repeatedly in conjunction with contact tracing, can prove valuable in formatting public policies and mass preventative measures during reopening phases [32,33]. Additionally, moving initial diagnosis for COVID-19 from a limited number to a large population can help understand community spread dynamics and disease history. That can, subsequently, facilitate obtaining the real statistics of the pandemic, providing better treatments and minimizing the long-term manifestations of infection. Altogether, these can be potentially transformative in formulating better predictive models and preventative policies to improve the quality of life during post COVID-19 stage.

Several companies and research groups are validating, producing, and marketing lateral flow antibody assays as rapid point-of-care tests to identify active COVID-19 cases [34]. However, the ability of antibody tests to accurately detect exposure to the severe acute respiratory syndrome coronavirus-2 (SARS-CoV-2) relies on their sensitivity and specificity, which can vary significantly between different assays. Furthermore, these tests are less likely to detect patients in the early stages of the disease. Despite these factors, the widespread use of serology tests as rapid point of care tests (PoCTs) could reveal the actual number of infections and infection mortality rates. These results can inform researchers of the complete disease statistics and help in designing better epidemiological models. This article presents a comprehensive review of the background and present developments in antibody-based mass testing. It also emphasizes the profound importance of developing robust and accurate tests and how judicious usage can serve the purpose of effective population surveillance and post-pandemic management.

## 2. The Relevance of Rapid Tests in a Pandemic Diagnosis and Surveillance

Diagnosis, as well as monitoring of diseases, are the essential factors for clinical management, especially during outbreaks. The primary necessity in a pandemic scenario understandably constitutes of easy, rapid, and field-deployable mass testing tools. The various attributes of quick testing approaches that make them relevant are:

(i) They can be accessed in rural and urban settings, developed, and developing countries alike, like a simple blood glucometer or pregnancy strip test.
(ii) They can be used for the mass diagnosis utilizing rapid tests that prevent patient leakage as diagnosis and following remediation steps can happen simultaneously.
(iii) They can significantly reduce the turnaround time for test results, which becomes crucial in a community transmissible disease outbreak.
(iv) They can be combined with biostatistical and bioinformatics analysis, which facilitate the quick recording of an enormous number of test results, data storage for easy reference and practical use for constant patient monitoring.

In past episodes of viral and non-viral contagious disease outbreaks, conventional methods such as biochemical assays, pathogen culturing, ELISA, RT-PCR, and other nucleic acid amplification tests (NAATs) [35] have played a significant role. Their major negative aspects are that they are time-consuming and expensive [36]. Moreover, such tests were not suitable to identify or track past infections for recognizing and mitigating the after-effects of the infection on a community basis. Nonetheless, during an ever-evolving transmissible viral pandemic, such as the current one, complete community-based initial diagnosis is not practicable, especially for asymptomatic patients. In view of that fact, antibody-based rapid tests can be the first line of practicable mass testing.

Compared to laboratory-based thorough testing protocols, which can be used for a greater accuracy on a shortlisted number of patients, a rapid test poses a considerable advantage. Rapid tests such as antibody-based ones also have a great prospect in mass surveillance or in past infection screening from a public health perspective [37]. 

## 3. Evolution of Antibody-Based Mass Testing

**Figure 2:**
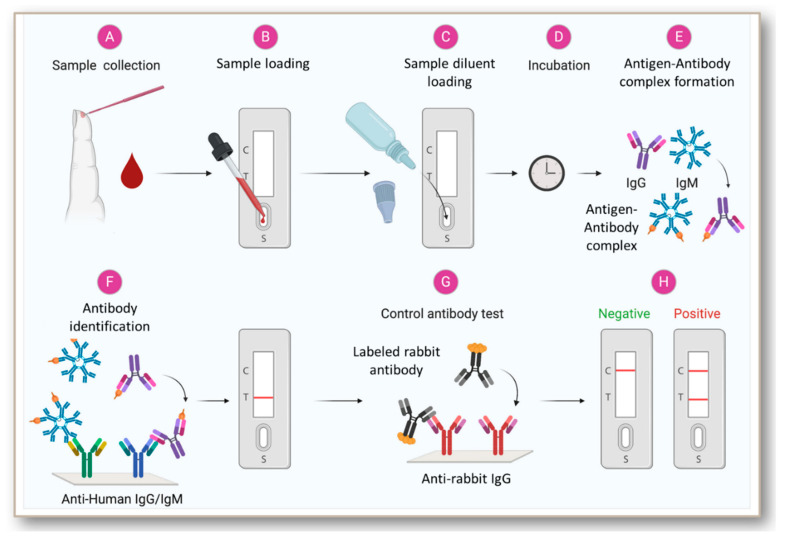
Scheme showing the general steps in the antibody-based diagnosis of viral infections from blood samples. Rapid antibody tests are performed using blood samples. After the addition of diluent buffer, the sample/diluent mixture flows down to the sample pad by capillary action/lateral flow and hits the conjugation pad. The conjugation pad contains the viral antigen conjugated to a specific label molecule or nanoparticles. During this stage, any antibodies in the sample with specificity for the virus will bind to the antigen and its conjugated label. Next, the sample/conjugate complex moves to the nitrocellulose membrane. Here, it meets the three test lines: IgG, IgM, and control. First is the M line, which contains an immobilized antibody that recognizes human IgM. Any IgM antibodies will bind here. However, only human IgM antibodies specific for the viral infection form antigen/label complexes which will produce a visible colored line. Second is the G Line, which contains an immobilized antibody that recognizes Human IgG. All IgG antibodies will bind here. However, only human IgG antibody/virus specific antigen/label complexes will produce a visible colored line. The control line is the last line the sample will encounter. The control line contains an immobilized antibody that recognizes Rabbit IgG, the control antibody. To serve as a procedural control, a colored line should always appear in the control line region, indicating that the proper volume of specimen has been added and membrane wicking has occurred. Generally, the results can be read after 10 min.

In 1917, Dochez and Avery were the first to report the utilization of immunoassay at a mass scale for the diagnosis of infectious diseases while testing for pneumococcal polysaccharide in patients with lobar pneumonia [38]. The introduction of radioimmunoassay (RIA) in 1960 and the ELISA in 1971 added higher sensitivity to the immunoassays for antigen or antibody-based disease diagnosis [39,40,41]. Subsequent early innovations comprised of immunoassays dependent on capillary migration in cellulose acetate sheets as a structural support, and coupling of antibodies to colloidal gold or latex [42,43]. Further, progress in this field resulted in the development of more advanced technologies such as the lateral flow immunoassay (LFIA) platform [44,45,46] (Figure 2). Sandwich immunoassays have been the basis for the development of rapid diagnostic tools for various infectious diseases [47,48,49,50]. The test kits for HIV-1/2 or hepatitis C virus follows a LFIA format that consists of a strip of an immobilized target antigen to which the patient antibodies bind [51,52,53,54,55]. A labeled reporter, such as a second targeted antibody is used for detecting the patient antibody.

Several tests obtained a Clinical Laboratory Improvement Amendments (CLIA) waiver that permitted their largescale use as rapid test tools and could be adopted even by the least developed nations [52,56]. The OraQuick Advance Rapid HIV-1/2 antibody test is an FDA approved test for HIV using a non-invasive, easy, at-home method for sample collection [52]. Similarly, the adoption of rapid tests for malaria diagnosis resulted in safeguarding the lives of hundreds of thousands of people, even in the most under-developed countries [57,58]. Another substantial improvement in antibody-based rapid diagnostic methods was the incorporation of nanotechnology-principles [59]. Nanomaterials have a high surface-to-volume ratio making them suitable candidates for target-specific attachment to the likes of antigen–antibody complexes, thereby increasing the sensitivity of tests [60,61]. The application of nano-diagnostics can hugely revolutionize the concept of accuracy and specificity in rapid antibody detection. Over the years, global health agencies have taken an increasing interest in infectious disease diagnosis using this robust, reproducible, and affordable means even in developing countries [61,62,63,64]. 

Despite shortcomings, antibody tests have the proven advantage over NAA- or antigen-based techniques in detecting otherwise hidden past infections within several weeks to few months. This can be immensely crucial to gauge the true infection numbers and more realistic disease statistics as it is difficult to diagnose all patients during their active infection phase. 

## 4. Antibody-Based Tests for SARS and MERS: A Basis for COVID-19 Testing

Antibody-based diagnosis have historically been used in the detection of viruses, including Ebola [28], Epstein-Barr [65], and Zika [66]. Immunoglobulin testing methods have also found applications in the diagnosis of several coronaviruses. Earlier, coronaviruses were associated with the common cold and other mild ailments in humans [67,68], until they took the form of aggressive outbreaks in the 21st century viz. severe acute respiratory syndrome (SARS) [69], Middle East respiratory syndrome (MERS) and currently COVID-19 [70] (World Health Organization, 2020). SARS-CoV and MERS-CoV, the causal viruses for SARS and MERS, respectively, were both zoonotic in origin just like SARS-CoV-2, and they followed similar etiologies, causing mass infections of epidemic proportions [71]. MERS, however, followed a sporadic zoonotic spread and had restricted chains of human spread [72]. Although MERS did not spread much beyond the Middle East and did not cause global panic, the mortality rate was reported to be ~35% [73]. This episode following SARS, indicated the possibility of recurrence of infectious human coronaviruses globally. In 2017, the World Health Organization (WHO) included SARS-CoV and MERS-CoV in its Priority Pathogen list, to promote scientific investigations and develop rapid mass diagnostic measures for similar outbreaks. Indeed, the countermeasures for SARS-CoV-2 largely utilized SARS-CoV and MERS-CoV as prototypes [74]. 

**Figure 3:**
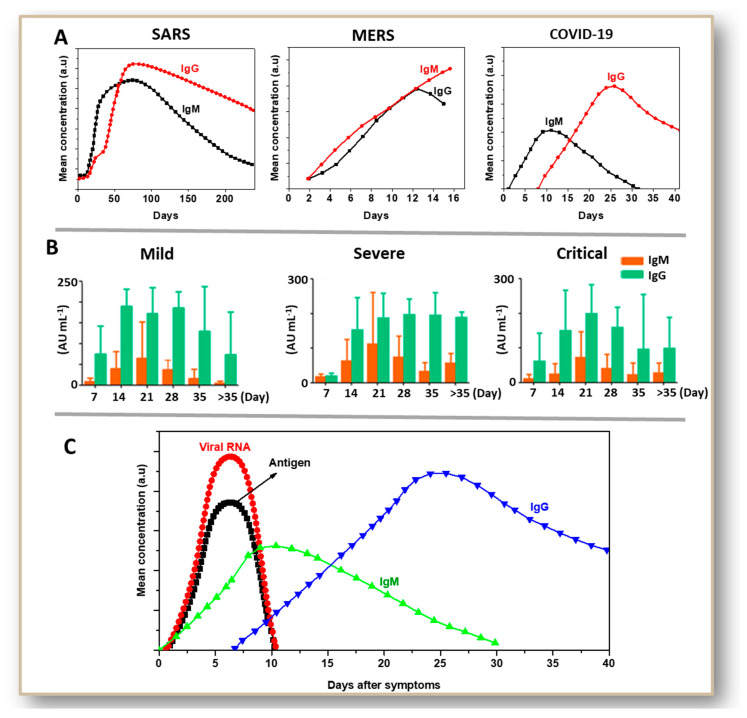
Immunoglobulin M (IgM) and Immunoglobulin G (IgG) serology profiles of SARS, MERS, and COVID-19, showing the time-dependent differential expression of these antibodies (**A**). IgM and IgG profiles of COVID-19 cases with mild, sever and critical conditions (**B**). Time-dependent presence of viral RNA/viral antigen in nasopharyngeal samples or immunoglobulins in serum of COVID-19 patients (**C**). Data for plotting graphs in A for SARS serology profile [82,83,84], MERS serology profile [85,86,87], and COVID-19 serology profile [88,89,90,91] are obtained from multiple research articles as indicated. Figure (**C**) shows the general trend indicating the presence of viral RNA, antigen (in nasopharyngeal samples) and antibodies (in blood) in a person’s body after SARS-CoV-2 infection. B is reproduced with creative commons attribution license (CC-BY-0.4) from reference [94].

All infections due to coronaviruses have some similarities at the level of immunological response (Figure 3A). During the acute phase of illness (1–7 days), detectable specific antibody responses against coronaviral infections were rarely observed [75,76,77]. Marked augmentations in antibody titers were observed after the second or third week of infection due to HCoV-229E, MERS-CoV, and SARS-CoV infections [78,79,80,81]. In case of SARS-CoV, a very long immunological response was observed. In symptomatic patients, there was a significant decrease in IgM antibody content after 150 days of symptoms [82,83,84], whereas IgG titer started to diminish after as late as 200 days. MERS serology studies revealed a similar profile between IgM and IgG [85,86,87]. A steady increase in both IgM and IgG concentration for 2–3 weeks was observed before a decline. In the case of COVID-19, IgM expression started between 3–6 days after infection and peaked during 9–15 days of the occurrence of symptoms [88,89,90,91]. IgG expression began after 10 days of symptoms and peaked between 20–30 days after the event of symptoms. IgM and IgG profiles considerably varied between acute, severe, and critical patients (Figure 3B). However, asymptomatic patients show significant variations in antibody profiles ranging from the lower level of expression to rapid clearance from the blood [92]. Over the years, healthcare experts have been dependent on both binding assays (ELISA, western blotting, immunofluorescence assay) and neutralization assays to detect the activities of different isotypes like IgM, IgG, and IgA (Immunoglobulin A). Clinically, IgM- and IgG-based assays are most relevant. Determining the time-window for symptom onset and detection of antibodies against major viral disease outbreaks of this century [93], have been big steps following the successful employment of antibody-based diagnosis. Soon after the occurrence of symptoms, both viral RNA and proteins (antigens), especially the spike protein, are detected in the nasopharyngeal samples (Figure 3C). Thus, NAATs and antigen tests are most effective during the first week of infection. In contrast, they cannot give reliable results as the viral shedding and antigen generation decreases after ten days. Although antibody production by the host’s immune system begins after a few days, antibody detections become more dependable after 14 days since infection onset.

In case of SARS-CoV, the primary antibody detection methods included ELISA, Indirect Immunofluorescence Assay (IFA), and neutralization tests (NT) [95,96]. ELISA can detect a blend of IgM and IgG antibodies formed within about 11 days of initial infection but involves a long protocol. IFA consists of binding of IgM and IgG antibodies present in the patient’s serum with specific labeled antigens or synthetic epitopes present in the test reagent. Subsequently formed antigen–antibody complexes can be visualized and imaged under a microscope [97,98]. NT is a quantitative test that titrates the neutralizing capability of patient sera [99], and can provide the best correlate of immunity. However, these tests can only be performed in biosafety level 3 (BSL 3) labs as they use viral particles. Pseudoparticle neutralization assays that use viral antigen mimicking nanoparticles can be performed without BSL 3 facility [100]. Furthermore, immunodot that is capable of detecting IgA, IgM, and IgG, on the same principle as western blotting using synthetic peptides and recombinant proteins, comes at a lower cost [79,101].

ELISA and microneutralization assays using MERS virus cultured in Vero cells showed a high degree of sensitivity and specificity [102,103] in detecting MERS virus too. On average, the antibodies to MERS-CoV virus were detected after 10 days of the onset of the disease. Clinical observations showed that around 42% of the results, which were tested negative for MERS-CoV by RT-PCR, were found to be MERS-positive by immunoglobulin based PoCT such as immunofluorescence assays and microneutralization [104]. Thus, combining antibody-based tests with NAA was a crucial step in MERS diagnosis. 

Despite the positive reports and advantages as described, no single antibody test kit could be universally approved and commercially available for mass-testing in either of the two outbreak episodes of SARS and MERS. This was primarily due to their inability to pass the threshold sensitivity and specificity requirement for better positive and negative predictive values. That, in fact can serve as a lesson, inducing wider collaborations and investments to achieve higher standards for SARS-CoV-2 antibody detection kits.

## 5. Development and Status of Immunoglobulin-Based Rapid Tests for COVID-19 

In the wake of a global crisis in late 2019 and evolution into the COVID-19 pandemic, a pressing need for a quick and reliable mass diagnosis method was felt by researchers, healthcare workers, and regulatory bodies around the world [105]. Based on existing knowledge and experiences during past viral outbreaks, the development of a scalable and rapid antibody test to be used for mass diagnosis and surveillance seemed like a logical approach. After SARS-CoV-2 enters the human body, it migrates to the respiratory tract, and finally infects alveolar type-II cells of lungs causing Acute Respiratory Distress Syndrome (ARDS) [106]. The immune system produces IgM, IgG, and neutralizing antibodies, which can block the virus from entering cells. Useful antibody tests are designed to detect the presence of SARS-CoV-2-specific antibodies in the blood [73,107,108,109,110]. 

The approaches that could be used for antibody-based COVID-19 detection are broad and are as follows:

- Binding antibody detection: these tests use specific reagents against individual isotypes such as IgM, IgG, IgA, and are tested against purified viral proteins in BSL2 laboratories.
  (a) The rapid tests or PoCTs utilize lateral flow devices for individual or combined antibody detection.
  (b) The laboratory assays are based on ELISA or chemiluminescent immunoassay (CIA).
- Neutralizing antibody assay: these tests involve infecting and incubating cultured cells with the virus isolated from patient samples, and then determining the functional ability of antibodies to prevent infections in vitro.
  (a) Virus neutralization tests use plaque reduction or microneutralization on clinical isolates.
  (b) Pseudovirus neutralization tests, on the contrary, use recombinant pseudoviruses.

When compared to rapid strip-based tests, the other ones are better in terms of specificity and accuracy. Still, they are limited by the need for either BSL2 or BSL3 facilities along with specialized tools. Lab-based tests also require trained personnel. Neutralization assays specifically are 4–5 days long procedures.

A recent study by Chen et al. demonstrated that among antibody responses against receptor-binding domain (RBD), spike glycoprotein type 1 (S1), ectodomain (ECD), and nucleoprotein (NP) [111] ECD- and S1-specific IgA show neutralizing potential. This study also indicated that simultaneous detection of NP-specific IgM and ECD-specific IgG could significantly improve the overall sensitivity. This combined effect was more apparent in the early phase of infection (first two weeks of symptom onset) which could be associated with development of an NP- or RBD-specific IgA immune response in the early phase of infection and late development of IgM and IgG responses [112,113]. The binding assays are directed towards the NP or S protein of SARS-CoV-2 virus, those epitopes being more or less conserved (primarily the receptor-binding domain or RBD of S-protein). There still can be some degree of cross-reactivity. Despite the fact that there can be false positives and false negatives, a rapid serology test, as long as it has a threshold specificity (>99.5%) and sensitivity (>90%), can serve the purpose of mass testing and surveillance according to regulatory bodies such as CDC. Many manufacturers are currently marketing COVID-19 antibody test kits for diagnostic purposes [114]. Considering test shortages, employers, health systems, and government bodies have been quick to buy. However, it is to be noted that the serological tests cannot serve as an independent diagnostic tool owing to possible false-negative results during early, yet contagious and symptomatic period, or in immuno-compromised individuals. Furthermore, since SARS-CoV-2 was a novel virus to invade humans, there was no existing data on the prognosis or host immunological responses to begin with. In addition to improving the quality of rapid tests and validating their accuracy to detect the SARS-CoV-2 antibody, a thorough understanding of the antibody dynamics was required. 

As reported by Irani et al., in a correspondence to Nature Medicine, published on 16 March 2020, the antibody-secreting cells (ASCs) registered an increase since day 7 after symptomatic presentation and were still detectable at convalescence on day 20 [115]. Activation of CD8+ T cells and CD4+ follicular T cells which is crucial for synergistic response to infection and inoculation by viral agents [116,117] was also observed. In this study, the singular non-severe, symptomatic patient showed resolution of symptoms at day 13 and was under observation till day 20. The patient’s IgM and IgG antibodies showed a progressive increase. In a similar index case study done in Finland, immunofluorescence assay (done on Vero E6 cells infected with SARS-CoV-2) showed the occurrence of neutralizing antibodies, including targeted IgM and IgG after 9 days. IgG titers showed an increase from 80 to 1280 between day nine and twenty from the onset of symptoms [118]. 

There are several studies that validated the sensitivity and specificity of various commercially available antibody COVID-19 tests [119]. Some of the important ones are given in Table 1. The findings from 34 hospitalized patients presenting an acute symptomatic phase of COVID-19 suggested that at least 32 patients showed a trend of increasing IgM and IgG up to a month after symptom onset, with IgM reaching the average highest expression after three weeks (322.80AU/ml, ref: <10 AU/mL) [89]. IgG production started following IgM, but that continued registering a steady presence (167.16 AU/mL) up until the last testing time point of seven weeks, while IgM expression declined after week four. In another study, published around the same time: at 5.5 days after initial symptoms, the detection efficiency of IgM via ELISA surpassed that of qPCR [120]. They combined the detection of IgA with the other two IgGs and found that it followed a similar expression pattern as IgM (initial detection ~5 days). At the same time, IgG was detectable at around the usual 14 days. The dynamic range of IgM and IgG was also quantified in serum samples of 85 confirmed (RT-PCR detectable) and 24 suspected patients of COVID-19 at Union Hospital, Tongji Medical College of China, between 3 and 40 days after symptom presentation [121]. These researchers employed a sandwich ELISA method with recombinant SARS-CoV-2 nucleocapsid protein. They detected an average consistency rate of more than 85% for IgM and IgG in both the lab-confirmed and suspected cases. 

In a letter to the editor published in Medical Virology, the authors reported that out of 60 patients, after convalescence, 13 patients had detectable IgG and undetectable IgM. Ten patients showed a decline in both IgM and IgG in a second test, one week after convalescence, correlative to negative RNA tests (both times) and improvement in chest CT-scans. The results supported serodiagnosis to be a robust indicator of the stage of COVID-19 [128]. In another cohort study, including 23 patients (median age 62 years) with lab-confirmed COVID-19, in two Hong Kong hospitals [137], the authors reiterated earlier reports that antibody assay can complement NAATs for diagnosis. They implemented and advocated the use of self-collection of posterior oropharyngeal swab samples to prevent the high exposure-risk of healthcare workers while collecting nasopharyngeal or throat swabs. The latter methods can lodge coughing or sneezing responses by the patients. They also tested for IgM and IgG antibodies against two different antigenic sites of the SARS-CoV-2 virus, nucleoprotein and spike protein, with similar trends in appearance and titers. Thus, serological tests can be crucial means of retrospective diagnosis, since microneutralization studies using recombinant spike protein, specifically, RBD, have shown prominent results for both IgM and IgG binding and specificity [71,138,139,140]. Thus, an effective antibody detection method should account for both the IgM and IgG antibodies and could also reduce the exposure of healthcare workers by utilizing blood collection.

In a report published on 13 April 2020, M.A. Al-Muharraqi of Royal Medical Services of Bahrain Defense Force, made an important suggestion of using serological testing as a complementary method to repeated testing for PCR detection. He suggested that to monitor patients admitted for non-COVID emergency surgeries, such as head and neck cancers, and to prevent postponing them. Even while keeping in mind the various paraphernalia of antibody detection, he recommended the use of this kind of test owing to its affordability and rapidity to monitor patients [141]. The same application can be extended to mass containment zones such as dormitories or hospices.

**Figure 4:**
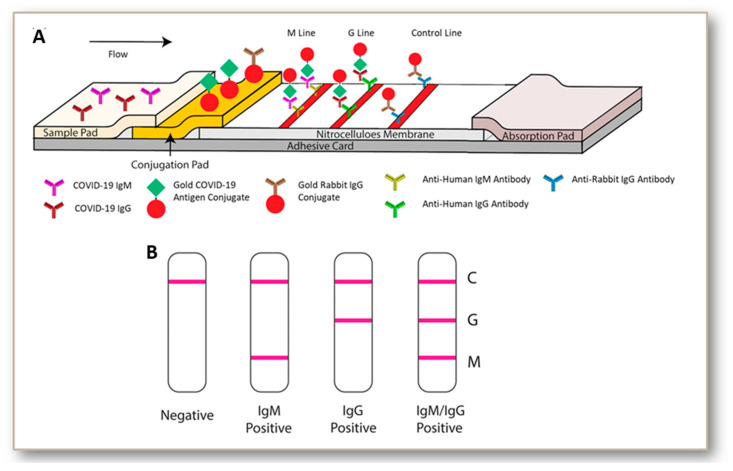
Steps in lateral flow immunoassay (LFIA)-based COVID-19 diagnosis (**A**). LFIA is performed by using the blood sample collected from the individual with suspected COVID-19 suspected infection. Lateral flow tests involve the migration of an antigen or antigen–antibody complexes, through an absorbent pad which is attached at the end of the strip. A liquid sample containing the analyte moves through the various zones of the polymeric strip. The sample pad acts as sponge and once soaked it allows the fluid to pass to the second conjugate pad. The conjugate pad contains all the reagents required to conduct a chemical reaction between antigen and antibody. As the antigen passes through the pad there forms a mark. The antigen continues to pass through the test and control lines. After passing through these lines, the fluid enters to a porous material which serves as a waste container. Depending upon the patient’s condition either negative (only control mark developed), IgM (+) (both IgM and control marks are developed), IgG (+) (both IgG and control marks are developed), or both IgM and IgG (+) (all the marks are developed) results are obtained (**B**). Reproduced from reference [114] with permission from Wiley.

A recent population screening study indicated that IgG tests targeting the S antigen (Euroimmun and Liaison) lacked sensitivity and specificity (<95%) in clinical samples from patients exposed to various viruses [142]. The S1 based IgA assay, in contrast, had a relatively good sensitivity [143]. When comparing the laboratory assays in patients with different severity and stages of disease (overall), more than 14 days post-onset, the RBD based Wantai ELISA had the best overall performance. This ELISA also showed a potential to set a threshold indicating the presence of protective antibodies [143]. Another study indicated that Augurix IgG rapid diagnostic kit displays a relatively high accuracy for SARS-CoV-2 IgG in high COVID-19 prevalence settings [144]. Such kits can be suggested for mass serology testing [144,145]. Among the many currently available antibody-based tests, the popular ones are lateral flow immunoassays (BioMedomics quick test and Surescreen rapid test cassette), time-resolved fluorescence immunoassays (Gold site diagnostics kit), and colloidal gold immunoassays (VivaDiag COVID-19 IgG-IgM test and Assay Genie rapid POC kit). All these assays involve pipetting a few drops of blood from a finger prick or vein onto the immunoassay device, followed by a couple of drops of buffer solution. The results are displayed within 10–15 min, as depicted in Figure 4. However, some of them including BioMedomics quick test have been temporarily withdrawn from US market due to the imposition of new policies in validation [146]. We also found a registered clinical trial protocol for VivaDiag and anticipate that further clinical accuracy data will become available as time progresses.

## 6. Challenges and Pitfalls 

There are two glaring problems with the tests based on antibody detection. Firstly, almost all of the COVID-19 antibody-based tests that have hit the market have been reported to be of questionable accuracy. Most are rapid qualitative tests that can be performed in doctors’ offices, workplaces, or homes. Unfortunately, none of them could match the reliability of lab-based thorough antibody tests [147]. Some recent reports suggest that nanoparticle-based lateral immunoassays would be considerably more accurate [148]. Nevertheless, there needs to be further rigorous research on the strip-based tests for at least one or a few of them to be authorized for unrestricted universal usage. The second limitation of antibody testing is a lack of data on immunity. Initial studies involving small groups of recovered COVID-19 patients suggest that short-term immunity may occur in some patients. However, long-term immunity is not definitively known or understood yet. Evidence on related coronaviruses is scarce and sometimes conflicting. More than one study suggest that immunity to SARS-CoV-2 might not last very long, some of them basing this theory on evidence of short-lasting immunity to seasonal coronaviruses [149]. Other studies indicate that immunity to the original SARS-CoV lasts for at least a few years but declines in many people [150]. Recovered patients also show differing immune responses [92]. The reason could be due to testing limitations and lack of data. However, it is also possible that people with milder disease symptoms spark weaker antibody responses. 

Some other factors must also be considered. A rapid serological test cannot serve as an independent diagnostic tool owing to several reasons. Detectable amounts of antibodies appear in the patient’s blood after a few days of the initial onset of symptoms. Although on average IgM appears in detectable quantity after about 5 days, based on variations reported, that can be as late at 11–12 days after infection onset [151,152,153]. Even in a 4–5-day window period, one can be asymptomatic but highly contagious and can infect other people in the close surroundings. Moreover, an effective antibody detection method should account for both the IgM and IgG antibodies, and they can vary widely between individuals. This makes it exceedingly challenging to standardize antibody detection approaches and to use them as a sole diagnostic tool [130,154]. A large cohort of immuno-compromised patients might give complete false-negative results, as they would not produce antibodies like normal individuals [130]. Considering the critical question of transmissibility timeline and serologically silent cases (specifically, in early yet active infections), rapid strip-based tests need to be used judiciously in entry/exit points of mass transits such as airlines. In many instances, airline passengers are screened using rapid antibody-based tests. However, there can be several COVID-19 active infections that go undetected. That can result in contamination of the plane, and community transmissions to co-passengers. Furthermore, antibody tests can be cross-reactive and can detect antibodies against other less severe seasonal coronaviruses to generate false-positives [113,155,156]. To resolve such cross-reactivity issues, it is necessary to design highly specific capture-antibodies against SARS-CoV-2 antibodies.

## 7. Significant Advantages and Prospects

Although the RT-PCR-based molecular test is specific and robust, it has its limitations such as the requirement of quality patient sample viz. nasopharyngeal swab, significant quantity of viral RNA, and chance of RNA degradation during storage and transportation. Largescale implementation of such tests can also be restricted by need of trained professionals for sampling, lab tests, etc. However, the most restrictive limitation could be the inability to detect past infections. In a typical scenario, all these factors can be addressed by strictly adhering to the SOPs (Standard Operating Procedures). Still, they generate financial and practicability issues during a pandemic. Whereas, despite its shortcomings, moving diagnosis for COVID-19 out from laboratory infrastructure can possibly be a breakthrough in mass testing and surveillance. Most of the investigations regarding the prognosis of COVID-19 and its antibody dynamics tell us that the viral load detectable by RT-PCR or antigen-based tests declines steadily after 9–10 days of initial infection [3,157,158], while IgM and IgG levels start peaking usually after a week [93,159,160]. Thus, the sole reliance on molecular diagnostics capable of detecting only active infections can cause serious under-detection similar to in China and the USA [161]. Antigen-tests are increasingly being considered more reliable, but they cannot possibly detect past infections after few weeks. Rapid antibody kits are being used to perform diagnosis without sending samples to centralized laboratories. This is helping communities without laboratory facilities identify active and past infections [36,156,162]. 

Although we have had earlier episodes of viral disease outbreaks such as SARS and MERS, none assumed pandemic proportions like the present. As COVID-19 is running rampant, researchers and healthcare workers have faced the significant challenge of getting a handle on the correct and complete disease statistics. A large number of cases going undetected have resulted in skewed statistics for the total number of infections, infection mortality rates, etc. Furthermore, since this aggressive invader resulted in many asymptomatic or pre-symptomatic patients, there is a big chance that not all individuals can be diagnosed at the active infection stage. Hence a mass diagnostic method based on immunoglobulins that persist in blood at least for several weeks to few months even after the infection is resolved is a logical approach to detect the otherwise hidden cases [163]. A retrospective diagnosis system such as antibody-based tests can undoubtedly serve the purpose of figuring out the correct numbers while generating more information about the transmission dynamics of this pandemic [164]. Numerous well-designed serosurveys utilizing antibody-tests are now being performed to this end [163,164,165]. These can help in developing epidemiological data and finally obtain the much-needed complete and correct etiology and statistics of COVID-19 to help build a better post-pandemic world [93,138]. In addition to serological tests being a practical candidate for 1st line of diagnosis, if the instructions can be standardized for rapid kits, then combined with an extensive set of questionnaires, these strips can be bought with prescription as a set of 3–4 tests and can be used at a gap of 5–7 days at home. 

There are several rapid antibody test devices in development that are potentially reliable as PoCT for the diagnosis of COVID-19. Recent commercially available lateral flow test kits such as Wondfo^®^ SARS-CoV-2 Antibody Test (Guangzhou Wondfo Biotech Co., Ltd., Guangzhou, China), Innovita^®^ 2019 n-CoV Ab Test Colloidal Gold (Biological Technology Co., Shanghai, China) and SGTi-flex COVID-19 IgM/IgG (Sugentech) also showed promising results. These are gold nanoparticle-based immuno-chromatographic test kits for qualitative determination of COVID-19’s IgM and IgG antibodies in whole human blood, serum, or plasma. Despite their limited reliability as a diagnostic tool, antibody testing using a relatively simple strip-based method (lateral flow chromatography) can be used to obtain qualitative results (presence or absence of detectable quantities of IgM/IgG) in about 15 min. However, chances of recurrent infections of COVID-19 cannot be entirely neglected. A combination of multiple tests, including sequence comparisons of viral strains involved in multiple episodes (in the case of recurrent infections), viral culture-based methods, and determining the innate/adaptive immunity, could unravel the challenges in identifying these recurrences [166]. Hence, combining PCR results with antibody detection can possibly be a reliable means of detecting recurrent infections [34]. 

Contextually, as one of the later developments in the COVID-19-related complications, MIS-C or multisystem inflammatory syndrome in children needs a special mention [167,168,169,170,171,172,173,174,175,176]. On 2 May 2020, a Pediatric Intensive Care-COVID-19 international collaborative conference call was held, where panelists recognized MIS-C as an emerging effect of COVID-19 infections in children [177]. Symptoms reported could range from two or more organ dysfunctions including cardiac, GI, renal, neurological, respiratory disorders, conjunctivitis, systemic shock, and systemic inflammation [178]. Since June 2020, similar symptoms have been reported in adults termed MIS-A, that were usually accompanied by respiratory failure. Notably, MIS-C or MIS-A being delayed manifestations of SARS-CoV-2 infection, a significant percentage of these patients were observed to be PCR- and antigen-negative, yet often antibody-positive, with varied accuracy [179,180]. These positive antibody detections could be due to persistent multiorgan dysfunction or multisystem inflammation even after active infection was resolved. These cases highlight the applicability of antibody-based tests to trace past COVID-19 infections, while performing contact tracing. 

Antibody testing also has a pronounced role in vaccine development. It is essential and important to make sure that the volunteers selected for vaccine trials are free from virus-specific antibodies in their blood to understand the formation of vaccine associated antibodies. Moreover, it is also necessary to perform periodic testing of volunteers who have received the vaccine to understand the effectiveness of the vaccination. Post immunization assessment of antibody levels, at least during the trial stage, is essential to decide the time window for booster shots. Antibody-based tests would be inevitable for assessing the efficacy of candidate vaccines by controlled human infection models (CHIMs) too [181]. Although there are concerns regarding the possible declining antibody levels in COVID-19 recovered patients, the concept of “immunity passports” is still an active area of discussion. Theoretically, certification of an individual with active immunity against the virus can minimize the burden of further testing before entering in a new geographical location where the entry is regulated. Periodic testing of antibodies against the virus is crucial to identify the level of immunity and provide or renew such certifications.

In addition to mass diagnosis, regulatory bodies are now employing immunoglobulin-based tests for surveillance and control. The Centers for Disease Control and Prevention (CDC), for example, have strategies in place to run mass antibody testing in multiple states of the USA on donated blood in blood banks. Simultaneously, they are planning mass antibody testing of different populations at different time and locations [182]. Several blood banks including the American Red Cross are offering free antibody-tests for COVID-19 to potential donors, clearly to serve the dual purpose of checking the status of donated blood and for past infection data of the donors. Apart from diagnostic benefits, immunoglobulin-based tests also help to identify potential donors of therapeutic plasma and provides crucial data and knowledge for vaccine development [183].

## 8. Conclusions

Accurate and scalable antibody-tests for COVID-19 would increase the scope for mass surveillance to be performed in the community. It can reduce the time to obtain an actionable result and can have important bearings on screenings, better pandemic statistics, and epidemiological data. Such information can help in understanding the long-term effects of infections in patients post-pandemic and boost future pandemic preparedness. Scientists, clinicians, and public healthcare professionals strongly believe that antibody testing using quality devices and with appropriate interpretation of the data are of real value amidst the hyperbolic or confusing media coverage on testing. If the application of this method is extended beyond its limited diagnostic applicability for the community testing of COVID-19, that will undeniably have a huge impact as a surveillance tool especially for understanding the level of immunity gained within a specific territory. Hence, in conclusion despite numerous pitfalls, the benefits and prospects of immunoglobulin-based diagnosis and surveillance bring great value to healthcare professionals, researchers, governments, and the public in this pandemic and post pandemic situation, alike.

## Figures and Tables

**Table 1:** Various antibody-based COVID-19 tests, their features, and available validation data.

Methodology Used for the Test	Main Findings	Number of Subjects	Categorization of Subjects	Sensitivity (%)	Specificity (%)	Positive Predictive Value	Negative Predictive Value	Reference
Lateral flow immunoassay (LFIA)	The IgM–IgG combined assay showed better sensitivity than a single IgM or IgG test.	525	397 were PCR positive and remaining 128 were negative	88.66	90.63	NA	NA	[114]
Chemiluminescence assay	Showed higher efficiency for the detection of IgM and IgG anti-SARS CoV-2 antibodies	125	61 were PCR positive patients and 64 were negative	73.3 (IgM) 76.7 (IgG)	92.2 100	81.5 NA	88.1 90.1	[122]
Chemiluminescence assay	IgG showed higher titre value than that of IgM	76	43 were PCR positive and the remaining 33 were probable cases	48.1 IgM 88.9 IgG	100 IgM 90.9 IgG	NA	NA	[123]
Chemiluminescence assay	Automated CLIA analyzer	176	125 PCR confirmed cases	95–95.5	100	100	NA	[124]
Chemiluminescence assay	Positive results after two weeks of symptom onset	34	All the subjects were PCR confirmed patients	94.1	NA	NA	NA	[89]
Chemiluminescence assay	Highest seroconversion was observed around 20–22 days after symptom onset	285	All the 285 were PCR positive patients	94 (IgM) 100 (IgG)	NA	NA	NA	[125]
Chemiluminescence assay	Rapid	476	276 PCR positive patients, and 200 negative volunteers	57.2 (IgM) 71.4 (IgG)	NA	NA	NA	[126]
ELISA	IgM and IgG were reliably positive after one month of symptom onset	24	All were PCR positive patients	74	100	NA	NA	[127]
ELISA	All patients tested positive for IgG and some patients tested negative for IgM	60	Samples collected from convalescent patients	78 IgM 100 IgG	NA	NA	NA	[128]
ELISA	The IgM reached 100% about one month of symptom onset	173	All the subjects were PCR positive patients	100 (>15 days)	NA	NA	NA	[112]
ELISA	IgA, IgM, and IgG were detected from 5 days from the onset of symptoms	140	82 PCR positive and 58 suspected cases	75.6 (IgM in confirmed cases) 93.1 (IgM in probable cases)	NA	NA	NA	[120]
ELISA	IgG and IgA ELISAs in along with EUROLabworkstation (Euroimmu)		39 PCR positive cases, 13 were IgG and IgA positive and 11 IgA only positive	NA	91.9% (IgG)73.0% (IgA)	NA	NA	[129]
IgM /IgG immunoassay	The rapid test	110	30 were PCR positive cases, 50 were persons with respiratory symptoms and 30 were PCR negatives	18.4	91.7	87.5	26.2	[130]
Immunochromatography	The IgM-positive rate showed an elevation from 11.1% in early-stage to 74.2% in late-stage disease. The positive rate of IgG in patients was 3.6% in early-stage and 96.8% in late-stage disease	105	105 patients	68.6	NA	NA	NA	[131]
Modified cytopathogenic assay	Patients with severe clinical manifestations showed higher antibody titre	70	The study comprises inpatients and Convalescent patients	100	NA	NA	NA	[132]
Immunofluorescence assay	Rapid and easy to use	59	59 suspected patients; 24 PCR positive cases	87.5	NA	NA	NA	[133]
Rapid immunoassay	The accuracy was 40% in the first week and 93.9% after 2	179	Patients included PCR positive (*n* = 90) and PCR negative (*n* = 89)	85.6	91	95.1	82.7	[134]
Rapid lateral flow assay	Fast, point of care test	67	67 were PCR positive patients	11 (<7 days) 92 (7–14 days) 96 (>14 days)	NA	NA	NA	[135]
Single Molecular Array (SIMOA)	Indicates disease severity	81	All were SARS-CoV-2 confirmed patients	86	100	NA	NA	[136]
